# Crossroads of Identity: Psychosis and Mental Health in Transgender Individuals

**DOI:** 10.1192/j.eurpsy.2026.12112

**Published:** 2026-07-31

**Authors:** M. Lavrador, D. G. Rodrigues, G. França

**Affiliations:** ULS Santo António, Porto, Portugal

## Abstract

**Introduction:**

Transgender individuals experience a higher prevalence of mental health disorders compared to cisgender populations, with emerging evidence indicating an increased risk for psychosis. Minority stress theory, which posits that chronic exposure to discrimination and social exclusion drives psychiatric vulnerability, is a central explanatory model.

**Objectives:**

This study explores epidemiological evidence on the prevalence and incidence of psychosis in transgender individuals, risk factors (including minority stress, neurodevelopmental comorbidities, and barriers to gender-affirming care), the impact of gender-affirming interventions on psychosis risk and mental health outcomes and identify gaps in the literature and propose future research directions.

**Methods:**

A comprehensive review of the literature focusing on transgender and nonbinary populations and psychosis or severe mental illness was conducted. Databases such as PubMed were searched for studies and reviews published from 2016 to 2025. Inclusion criteria encompassed registry-based cohort studies, systematic reviews and narrative syntheses.

**Results:**

Registry-based cohort data from the Netherlands demonstrated that transgender persons have a significantly increased incidence rate ratio (IRR) for non-affective psychotic disorders compared to matched cisgender controls, with IRRs ranging from 2.00 to 22.15 depending on recruitment method. Systematic reviews consistently report elevated rates of psychiatric morbidity, including psychosis, in transgender populations, though robust prevalence estimates remain limited due to methodological heterogeneity and small sample sizes. Minority stress, particularly gender-related discrimination, is the most documented risk factor, with both distal and proximal stressors contributing to poor mental health outcomes. Neurodevelopmental comorbidities, such as autism spectrum conditions, are more prevalent in transgender populations and may further increase vulnerability. Access to gender-affirming care is associated with improved mental health outcomes, while barriers exacerbate risk. Resilience-promoting factors include social support, self-esteem, and family acceptance.

**Conclusions:**

Transgender individuals are at increased risk for psychosis relative to cisgender peers, with minority stress, neurodevelopmental comorbidities, and barriers to gender-affirming care as key mediators. While the evidence base is growing, further research using representative samples and longitudinal designs is needed to clarify prevalence, mechanisms, and effective interventions.

**Disclosure of Interest:**

None Declared

